# Sex Differences in Resistance Training Participation and Beliefs Among Adolescent Athletes: An Exploratory Cross-Sectional Study

**DOI:** 10.3390/sports14010004

**Published:** 2026-01-01

**Authors:** Corey Marie Rovzar, David Howell, Aubrey Armento

**Affiliations:** 1 Department of Medicine, Stanford Prevention Research Center, Stanford University School of Medicine, Palo Alto, CA 94304, USA; 2 Department of Orthopedics, University of Colorado School of Medicine, Aurora, CO 80045, USA; david.howell@cuanschutz.edu; 3 Sports Medicine Center, Children’s Hospital Colorado, Aurora, CO 80045, USA; aubrey.armento@childrenscolorado.org

**Keywords:** strength training, resistance training, exercise, youth, athlete, adolescent, beliefs, injury prevention, sex differences

## Abstract

Background: Resistance training (RT) is widely recommended for adolescent athletes to enhance performance and reduce injury risk. However, sex differences in RT participation and beliefs during adolescence remain underexplored. Methods: This exploratory cross-sectional survey included 108 adolescent athletes (62 females, 46 males; ages 13–18 years) recruited from sports medicine and physical therapy clinics. Participants completed a 29-item questionnaire assessing demographics, sport involvement, and RT participation and beliefs. Items included RT frequency, duration, equipment use, age of initiation, and enjoyment. Statistical comparisons between sexes were conducted using chi-square tests for categorical variables and independent-samples *t*-tests for continuous variables, with significance set at *p* < 0.05. Results: Overall, 71% reported regular RT participation. Participation was higher in males than females but did not reach statistical significance t) (80% vs. 65%, *p* = 0.07). Females reported beginning RT at a younger age than males (12.1 vs. 13.4 years, *p* = 0.01). No significant sex differences were found in RT frequency (3.1 vs. 3.5 sessions/week, *p* = 0.33) or session duration (56.3 vs. 68.8 min, *p* = 0.17). Males reported greater use of barbells (70% vs. 43%; *p* = 0.02) and weight machines (87% vs. 57.5%; *p* < 0.01). Females reported significantly lower enjoyment of RT compared to males (48% vs. 70%, *p* = 0.02). Conclusions: This study describes sex-based differences in RT participation and beliefs among a convenience sample of adolescent athletes recruited from sports medicine and physical therapy clinics. Future research should prioritize developing and validating questionnaires to more accurately assess RT participation and beliefs and to guide efforts aimed at fostering positive, equitable training opportunities. Recruiting from a population outside of a clinic environment will enhance the generalizability of these findings.

## 1. Introduction

Resistance training (RT) has gained widespread support as an essential component of youth athlete development, helping to reduce sports-related injuries and enhance overall physical well-being and sports performance [1,2,3,4,5]. The American Academy of Pediatrics and other sports medicine organizations support the inclusion of RT in youth sports, emphasizing its safety and efficacy when properly programmed, executed, and supervised [3,6]. Reflecting this consensus, the World Health Organization updated its 2020 physical activity guidelines to recommend muscle-strengthening activities at least three times per week for children and adolescents [7].

RT is a form of conditioning involving an individual working against resistive loads for the purposes of improved fitness, health, and/or performance [1,8]. The resistive loads can include one or a combination of body weight, weight machines, free weights, resistance bands, and medicine balls [9,10]. For adolescent athletes, RT has been documented as a method to improve overall strength, power, running and sport-specific speed, and motor control [4,11,12].

Although once believed to be dangerous for youth due to the potential damage to growth plates, increasing evidence over the past two decades has identified an association between supervised participation in RT and a reduction in sports-related injuries and greater enjoyment and confidence in engaging in physical activity [13]. Both the International Olympic Committee (IOC) and National Strength and Conditioning Association (NSCA) have issued position statements supporting youth RT participation and highlighting its role in fostering lifelong positive exercise behaviors [9,13]. Injuries associated with RT typically result from improper handling of weights or poor exercise technique under excessive load. These risks are readily mitigated through professional supervision and instruction [1].

Despite broad support and documented benefits, regular RT participation among adolescents remains low worldwide [14]. Studies among adult populations have identified disparities in RT participation associated with educational attainment, health status, and sex, with those who are educated, healthy, or male more likely to engage in RT than those who are uneducated, have underlying chronic disease, or female [15,16,17,18]. We report sex differences because our survey assessed sex; however, observed differences may reflect gender-related social influences. Gender-based stigmas (e.g., beliefs that women who are muscular are not attractive) and misconceptions (e.g., that RT will make women look “bulky”) about RT have been shown to disproportionately limit participation among women [19]. Together, these epidemiological patterns suggest that social and structural factors contribute to sex differences in RT participation, potentially through gender-related norms and opportunities. From a social-cognitive perspective, gendered socialization processes, including modeling, reinforcement, and the development of self-efficacy, may shape adolescents’ perceptions of and engagement with RT, contributing to patterned differences in participation and beliefs by gender [20]. Moreover, task-specific self-efficacy and perceived enjoyment are established correlates of physical activity behavior that may be relevant to RT contexts [21].

While these factors have been examined in adults, little is known regarding participation in, enjoyment of, and beliefs about RT among youth athletes, and particularly, how these vary by sex and gender. This is of particular importance as female youth athletes have been identified as an at-risk population for increased injury rates relative to male youth athletes [13]. To address this gap, we conducted a cross-sectional survey of adolescent athletes to examine sex differences in RT participation, beliefs about RT, and enjoyment of RT.

## 2. Materials and Methods

### 2.1. Participants

This study received approval from the local Institutional Review Board (IRB) prior to participant recruitment and data collection. We included 13–18 year old male and female athletes who presented to a hospital-affiliated sports medicine center, outpatient physical therapy clinics, or who were active in local high school or club sport partnerships. Inclusion criteria included between the ages of 13–18 and participating in recreational or competitive sports. Recruitment was conducted via distribution of a flyer with a QR code that the participant could scan and then complete the electronic questionnaires. Flyers were posted and/or provided in sports medicine clinics, physical therapy gyms, high school training rooms, and via email listservs to high school and club sport partnerships. An Institutional Review Board-approved electronic informed consent form was used to obtain assent/consent from participants and a parent/legal guardian (if the participant was under the age of 18 years) before they completed the electronic questionnaire. Data was collected between April 2021–December 2021. Participation was voluntary and compensation was not provided for study participation.

### 2.2. Study Design

We conducted a cross-sectional study using an electronic RedCap questionnaire [22] to measure participation in RT and beliefs about RT among youth athletes. The survey included a total of 29 questions: 8 questions on demographics (sex, age, race, ethnicity, school grade, zip code, and current injury status), 4 questions on sports participation and specialization, 6 questions on family affluence, and 11 questions on participation in and beliefs about RT (Supplementary File S1). Sex was recorded as female or male based on participant self-report. Gender identity was not assessed in this study to reduce participant burden. To assess socioeconomic status, we used the Family Affluence Scale, a 6-item validated questionnaire scored from 0 to 13, designed to assess the socioeconomic status of the family based on an assets approach [23]. Scores of 0–7 indicate low affluence, 8–11 moderate affluence, and 12–13 high affluence. We assessed sports participation by asking “How many different organized sports (with a coach and structured practices and competitions) do you participate in?” We used a sports specialization scale [24] to assess the level of sports specialization from low, moderate, to highly specialized, with scores ranging from 0 to 6. Scores of 0–1 were classified as low specialization, scores of 2–4 as moderate specialization, and scores of 5–6 as high specialization.

### 2.3. Questionnaire Development

A custom questionnaire was developed specifically for this study to assess resistance training participation, enjoyment, and beliefs among adolescent athletes (Supplementary File S1), as no validated questionnaires existed at the time to measure the desired outcomes. Participants were instructed: “Please answer the following questions based on your training habits before coming to physical therapy. Do not include your physical therapy home program in your answers.” Thus, RT participation/volume items reflected pre-physical therapy training habits. RT questions included assessment of whether the athlete participated in RT, the frequency of RT per month, type of equipment used, seasonal frequency, access to weights, and beliefs about RT (e.g., RT will make me better at my sport). Questions related to family support and barriers to participation were also included and are provided in Supplementary File S1 but were not analyzed in this manuscript. Items on specific equipment used were displayed only to participants who reported engaging in RT. Denominators for equipment-use variables therefore reflect the RT subset (female *n* = 40, male *n* = 37). Belief items were administered to all participants. No formal content validation, pilot testing, test–retest reliability assessment, or psychometric analyses (e.g., internal consistency) were performed. The full questionnaire is provided in Supplementary File S1.

### 2.4. Data Analysis

Data are presented as mean (standard deviation) for continuous variables and the number within group (corresponding percentage) for categorical variables. We compared demographic characteristics, resistance training volume/timing/beliefs, and types of equipment used between female and male participants. Categorical variables were analyzed using chi-square tests when expected cell counts were ≥5 [25]. When expected cell counts were <5, Fisher’s exact test was used to provide a more accurate estimate of statistical significance [26]. For continuous outcomes, we calculated Cohen’s d from the independent-samples *t*-tests using the pooled standard deviation [27]. For categorical (2 × 2) comparisons, we reported φ (phi) [27]. For categorical tables with >2 levels (e.g., seasonality), we reported Cramér’s V [28]. As general guidance for interpretation, we used Chen’s thresholds for d (≈0.20 small, 0.50 medium, 0.80 large) and Cohen’s thresholds for φ/V (≈0.10 small, 0.30 medium, 0.50 large) [29]. Statistical significance was defined a priori as *p* < 0.05, and all tests were 2-sided. Statistical analysis was performed using Stata Statistical Software: Version 18 (StataCorp, LLC, College Station, TX, USA).

## 3. Results

### 3.1. Demographics and Sports Participation

A total of 158 individuals completed the consent form. Of those, 108 (68%) completed the questionnaires—62 females, mean age = 15.0 ± 1.2 years; 46 males, mean age = 14.9 ± 2.6 years—who were included in our analysis. There were no significant differences between female and male participants for demographic characteristics, family affluence, sport participation (total number of sports played), or sport specialization level (Table 1). Family Affluence Scale scores indicated predominantly moderate-to-high affluence in this clinic-recruited sample. More than half of participants reported being currently injured at assessment (overall 63/108, 58%).

### 3.2. Sex Differences in Resistance Training Participation and Beliefs

The majority of the study sample (71% of all participants) engaged in RT with a non-significant trend toward greater RT participation among males compared with females (80% vs. 65%; *p* = 0.07). Females reported beginning RT at a significantly younger age compared to males (12.1 vs. 13.4 years; *p* < 0.01). We did not observe any significant sex-related differences in the mean duration or frequency of RT between groups (Table 2). The majority of both the female and male participants engaged in RT both during the season and the off-season (Table 2).

Female participants reported enjoying RT significantly less than males (48% vs. 70%; *p* = 0.02, Table 2). No other significant differences regarding beliefs about RT were identified. Most female and male participants believed that RT would make them better at their sport, would help them prevent injuries, and felt comfortable lifting weights (Table 2). A small proportion of female and male participants believed that RT would result in looking bulky or with muscles that are too big (6% and 9%, respectively) or that RT will cause injuries (2%), with a greater proportion believing that RT will make them look good (35% and 50%, respectively, Table 2).

Equipment-use percentages are reported among those who engaged in RT (female *n* = 40; male *n* = 37). Male participants were significantly more likely than female participants to report using barbells (70% vs. 43%; *p* = 0.02) and weight machines (87% vs. 57.5%; *p* < 0.01, Table 3). There were no sex-differences observed in body weight, resistance band or cord, or free weight utilization. For male participants, the highest proportion utilized free weights, followed by weight machines, body weight, resistance bands or cords, and lastly, barbell (Table 3). For female participants, the highest proportion used body weight and resistance bands or cords, followed by free weights, weight machines, and lastly, barbell (Table 3).

## 4. Discussion

To our knowledge, this is the first study to examine sex differences in RT participation and beliefs among adolescent athletes. Overall participation in RT for both male and female athletes in our sample was higher than a previously reported estimate of high school students achieving the recommended US guidelines of muscle-strengthening activities three times per week (52%) [30,31,32]. The higher RT participation observed in this study may reflect our sports medicine and physical therapy clinic-based recruitment of recreational and competitive athletes, which likely increased participant exposure to RT through injury rehabilitation. Although participants were instructed to report pre-physical therapy training habits, prior injury and rehabilitation experiences may still have shaped their beliefs, contributing to selection bias and limiting external validity. Lastly, our measure of RT participation did not limit the analysis to those engaging in fewer than three days per week, although the mean frequency for both sexes was approximately three days weekly.

The majority of female and male participants believed that RT would make them better at their sport, help prevent injury, and were comfortable performing RT. However, females reported lower enjoyment of RT than males. This pattern may reflect a lack of social support, social stigma, the gym environment, a lack of confidence in skills, or different goals and motivation [19]. Lower enjoyment among females may also relate to their lower participation in RT relative to males, though causality cannot be inferred. Notably, despite strong beliefs about RT’s benefits, a gap between beliefs and behavior was evident with 87% of females endorsing RT benefits, yet only 65% participating in RT. This discrepancy underscores that positive beliefs alone may be insufficient and highlights the importance of addressing enjoyment, confidence, access, and social influences to translate intentions into behavior [33]. These patterns may be consistent with gender role socialization theory, which proposes that gendered norms and social reinforcement shape adolescents’ activity preferences [20]. Prior work suggests that social norms and training environments may influence girls’ comfort with RT settings [20]. In parallel, Social Cognitive Theory highlights the role of task-specific self-efficacy [21]. Lower confidence with RT skills or environments could coincide with lower enjoyment and participation. We note that these are interpretive frameworks rather than causal inferences, and longitudinal work is needed to test these pathways. In addition, body appearance pressures may represent another pathway shaping girls’ engagement with RT. Adolescent girls are more vulnerable than boys to sociocultural pressures to attain a lean, toned physique and to engage in appearance-motivated exercise behaviors [34]. Although we did not assess body image or social media exposure in this study, such pressures could influence RT engagement and motivations and affect the decision to use bodyweight or specific equipment promoted to achieve a lean versus bulky appearance. Future research should identify modifiable drivers of enjoyment and engagement to better support RT participation among adolescent athletes.

Although we observed no significant sex differences in RT participation frequency or duration, there were significant differences in equipment usage, with male athletes more frequently utilizing barbells and weight machines. While we did not ask participants to report their gender, these findings align with existing literature highlighting sex differences in equipment preferences, potentially influenced by perceived gender roles, access, and exposure to specific types of equipment [19,35,36,37]. Accordingly, incorporating the use of free weights, barbells, and weight machines into strength programming among young female athletes, with adequate instruction and supervision to promote comfort and confidence, may be beneficial.

Interestingly, the female participants reported initiating RT at a younger age than the male participants. Given evidence that boys typically engage in RT at higher rates than girls, partly reflecting societal norms and encouragement for strength-focused activities [38], one might expect earlier initiation among boys. Our findings may instead reflect earlier exposure for girls via rehabilitation, organized sport requirements, or greater uptake of injury-prevention programming in girls’ sports. This aligns with evidence suggesting that coaches of girls’ teams were more likely to be aware of and to adopt injury prevention programs than coaches of boys’ teams potentially due to greater public awareness of higher injury risk among girls for non-contact ACL injuries [39]. Understanding these distinct entry pathways into RT may help inform strategies to promote safe, enjoyable, and sustained RT participation across adolescence.

This study has several limitations. The survey-based method employed is subject to bias and more limited in assessing the nuances of participants’ participation in and beliefs about RT. All measures were self-reported and therefore may be affected by recall and social desirability bias, and we did not collect objective measures of RT frequency, duration, or equipment use. Generalizability is limited by our convenience, clinic-based sample from a single geographic region, with many participants presenting for sports medicine care or rehabilitation and from moderate-to-high affluence, which may restrict broader applicability. Further, over half of the participants reported that they currently had an injury. Although participants were asked to answer questions based on pre-physical therapy habits, prior rehabilitation experiences may still have influenced their answers. We did not collect data on the specific sports or competitive levels of the participants, which limits our ability to contextualize findings by sport type. In addition, we used a study-specific, non-validated 29-item questionnaire and did not perform formal content/face validation, pilot testing, test–retest reliability, or psychometric evaluation (e.g., internal consistency). This is a major methodological limitation that supports the exploratory, hypothesis-generating nature of the study. Our survey did not assess gender identity or expression and findings are limited to sex-based comparisons. Some observed differences may reflect gendered experiences and socialization rather than biological sex and future work should include validated measures of gender identity and related constructs. Finally, we are unable to report the response rate because we do not have the total number of people with access to the flyer/survey link which was posted in sports medicine and physical therapy clinics. This further limits our ability to assess potential selection bias. Despite these limitations, this is the first study to examine sex differences in participation in and beliefs about RT among youth athletes. The findings provide relevant information for coaches and sports medicine professionals to inform their approach to implementing RT among adolescent athletes, while taking into consideration sex differences. Future studies should investigate psychosocial factors, such as perceived competence, social support, and motivational climate, to better understand these sex-differences in RT attitudes and behaviors in a larger and more geographically and racially/ethnically diverse sample.

Resistance training (RT) is a critical component of youth athletic development, linked to improved sport performance and reduced injury risk [2]. This study provides important insights for coaches, athletic trainers, and strength professionals working with adolescent athletes. While overall RT participation was relatively high among both male and female athletes, key sex differences emerged as potential considerations for program design. Males were more likely to use traditional RT equipment (barbells and machines) and to report enjoying RT. These differences may stem from varied access, confidence, social influences, or past exposure to training environments [16,19]. Practitioners should consider these preferences when designing programs for female athletes. Creating supportive, inclusive training environments that emphasize competence and enjoyment may encourage greater engagement [40]. Coaches might start with bodyweight and resistance band exercises, gradually introducing free weights and machines as confidence builds [41]. Female athletes may also benefit from female role models, individualized instruction, and small-group formats that reduce intimidation [35,42,43,44]. Importantly, in our study, most athletes regardless of sex believed that RT improves sport performance and helps prevent injury. These shared beliefs can be leveraged to motivate participation. Tailoring communication strategies to reinforce these benefits may be especially effective for less experienced or reluctant participants [19,45]. In sum, practitioners can promote RT participation among youth by recognizing sex-specific preferences, enhancing enjoyment, and fostering confidence through progressive, supportive programming [1,9]. These efforts may encourage long-term adherence to RT and contribute to improved athletic development and injury prevention for all youth athletes.

## 5. Conclusions

This study is the first to explore sex differences in both participation and beliefs around resistance training among adolescent athletes in a clinic-recruited sample. Despite similar training frequency, female athletes reported lower enjoyment and less use of traditional weight equipment compared to males. These insights highlight the importance of addressing sex-specific preferences and perceptions to promote equitable, engaging resistance training experiences for all youth athletes. Future work should develop validated instruments across broader, non-clinical samples to provide greater evidence of the sex-differences observed in this study.

## Figures and Tables

**Table 1:** Study sample demographic characteristics by sex.

Variable		Female (*n* = 62)	Male (*n* = 46)	*p* Value
Age (years), mean (SD)		15.0 (1.2)	14.9 (2.6)	0.71
Race (n, %)	American Indian or Alaska Native	1 (2)	0 (0)	0.77
	Asian	2 (3)	1 (2)	
	Black Or African American	2 (3)	0 (0)	
	White	47 (76)	40 (87)	
	More Than One Race	7 (11)	3 (7)	
	Unknown Or Not Reported	3 (5)	2 (4)	
Ethnicity, n (%)	Hispanic Or Latino	10 (16)	8 (17)	0.59
	Not Hispanic or Latino	44 (71)	35 (76)	
	Unknown	8 (13)	3 (7)	
School Grade, n (%)	8	9 (15)	4 (9)	0.21
	9	17 (27)	12 (26)	
	10	23 (37)	12 (26)	
	11	7 (11)	13 (28)	
	12	6 (10)	5 (11)	
Sport Specialization Level, n (%)	Low	13 (21)	10 (22)	
	Moderate	25 (40)	12 (26)	0.27
	High	24 (39)	24 (52)	
Number of different sports played, mean (SD)	*-*	1.5 (0.8)	1.6 (0.8)	0.25
Currently Injured at Time of Assessment, n (%)	*-*	33 (54)	30 (65)	0.25

SD = standard deviation.

**Table 2:** Resistance training participation, volume, timing, and beliefs by sex.

Variable		Female (*n* = 62)	Male (*n* = 46)	*p* Value	Cohen’s d or φ Value
Perform resistance training exercises (yes), n (%)		40 (65)	37 (80)	0.07	Φ = 0.17
Age began resistance training (years), mean (SD)		12.1 (2.2)	13.4 (1.7)	<0.01	d = 0.68
Resistance training frequency (times per week), mean (SD)		3.1 (1.6)	3.5 (1.3)	0.33	d = 0.23
Resistance training duration (mins per session), mean (SD)		56.3 (44.8)	68.8 (30.0)	0.17	d = 0.32
Seasonality of typical resistance training, n (%)	During season	2 (5)	2 (6)	>0.99	
	During off-season	5 (13)	5 (14)		Φ = 0.02
	Both	32 (82)	28 (80)		
Believes resistance training will make me better at sport (yes), n (%)		54 (87)	42 (91)	0.49	Φ = 0.07
Believes resistance training will help me prevent injuries (yes), n (%)		48 (77)	33 (72)	0.50	Φ = 0.06
Believes resistance training will result in looking bulky or with muscles that are too big (yes), n (%)		4 (6)	4 (9)	0.66	Φ = 0.04
Believes resistance training will make me look good (yes), n (%)		22 (35)	23 (50)	0.13	Φ = 0.15
Believes resistance training will cause injuries (yes), n (%)		1 (2)	1 (2)	>0.99	Φ = 0.02
Enjoys resistance training (yes), n (%)		30 (48)	32 (70)	0.02	Φ = 0.21
Feels comfortable lifting weights (yes), n (%)		37 (60)	33 (72)	0.19	Φ = 0.12

**Table 3:** Type of equipment used by females and males who reported performing resistance training (n = 77).

Equipment Reported Using n, (%)	Female (*n* = 40)	Male (*n* = 37)	*p* Value	φ Value
Body weight	35 (88)	31 (84)	0.89	0.11
Resistance bands or cords	35 (88)	27 (73)	0.20	0.02
Barbell	17 (43)	26 (70)	0.02	0.29
Weight machines	23 (57.5)	32 (87)	<0.01	0.32
Free weights (dumbbells, kettlebells)	34 (85)	34 (92)	0.56	0.20

## Data Availability

The datasets used and/or analyzed during the current study are available from the corresponding author on reasonable request.

## Supplementary Materials

The following supporting information can be downloaded at: https://www.mdpi.com/article/10.3390/sports14010004/s1, File S1: Full questionnaire.

## Abbreviations

The following abbreviations are used in this manuscript:

RT	Resistance training
IOC	International Olympic Committee (IOC)
NSCA	National Strength and Conditioning Association (NSCA)
