# Supplementary material for: Sex Differences in Resistance Training Participation and Beliefs Among Adolescent Athletes: An Exploratory Cross-Sectional Study
Source: Sports (Basel). 2026 Jan 1;14(1):4. doi: 10.3390/sports14010004 (PMC12845955; doi:10.3390/sports14010004)
Supplement: Supplementary file 1 [file sports-14-00004-s001.zip › sports-3926358-supplementary.pdf]

**Study Questionnaire**

**Socioeconomic Questions**

1. Does your family have a car, van, or truck?
    - Yes, one
    - Yes, two or more
    - No

2. Do you have your own bedroom for yourself?
    - Yes
    - No

3. How many times did you travel abroad for holiday/vacation last year?
   *Because of COVID-19 this may be different from normal. Think about the last normal year you were able to travel.*
    - Not at all
    - Once
    - Twice
    - More than twice

   *Note: "abroad" defined as outside of the United States.*

4. How many computers does your family own?
    - None
    - One
    - Two
    - More than two

5. Do you have a dishwasher at home?
    - Yes
    - No

6. How many bathrooms (rooms with a bath or shower) are in your home?
    - None
    - One
    - Two
    - More than two

**Sport Participation**

*When answering sport participation questions, think about a normal year where COVID-19 restrictions did not cancel games or practices.*

1. How many DIFFERENT organized sports (with a coach and structured practices and competitions) do you participate in?

   (Do not include sports that you have recently quit.)

   - 1
   - 2
   - 3
   - 4

If more than 4 organized sports, answer the following questions about the four you care most about.

**Sports Specialization Questions**

For each item, response options:

- Yes
- No
- I do not have a primary sport

2. Do you consider your primary sport more important than other sports?

3. Do you play or train for a single sport more than 8 months a year?

4. Have you ever quit a sport to focus on a single sport?

**Resistance Training:**

Answer the following questions using the definition of resistance training below:

"Resistance training is exercise that involves using body weight, weight machines, free weights, resistance bands, and medicine balls. Examples include squats, deadlifts, lunges, shoulder press, bicep curls, chest press, push-ups, pull-ups, box jumps, planks, etc."

*Please answer the following questions based on your training habits BEFORE coming to physical therapy. Do not include your physical therapy home program in your answers.*

1. Do you perform strength exercises involving the use of body weight, weight machines, free weights, resistance bands, or medicine balls?
    a. Yes
    b. No

2. If yes, how older were you when you started resistance training?

3. If yes, do you participate in resistance training during season, during the off-season, or both?
    a. During season
    b. During the off-season
    c. Both

4. If yes, approximately, how many times do you perform resistance training per **week**?
    a. Fill in answer

5. If yes, approximately, how long (in minutes) do you spend per resistance training session?
    a. Fill in answer

6. If yes, what equipment do you use? (check all that apply)
    - Body weight
    - Resistance bands or cords
    - Barbell
    - Weight machines
    - Free weights (dumbbells, kettlebells)

7. If yes, when do you perform resistance training during the year? (check all that apply)

- Summer
- Fall
- Winter
- Spring
- Year-round

8. If yes, where do you have access to weights? (check all that apply):
    - No access
    - High school
    - Gym (fitness center or recreation center)
    - Home

9. If yes, does your family support your participation in resistance training
    a. Yes
    b. No
    c. Not sure

10. What are your beliefs about resistance training? (check all that apply):*
    - I believe resistance training will make me better at my sport
    - I believe resistance training will help prevent injuries while playing my sport
    - I believe resistance training will make me bulky or my muscles too big
    - I believe resistance training will make me look good
    - I believe resistance training will cause injuries
    - I enjoy resistance training
    - I feel comfortable lifting weights
    - Other beliefs about resistance training: please describe

*Asked to all participants (those who responded yes and no to participating in resistance training)

11. If you do not participate in resistance training, why not? (check all that apply):
    - I do not have enough time for resistance training
    - No one has taught me how to do resistance training
    - I do not know enough about resistance training
    - I do not have access to equipment
    - I have no one to do resistance training with
    - I do not want to do resistance training
    - I do not feel comfortable performing resistance training
    - I do not think that resistance training will help me in my sport
    - Other: please describe

If no,
